# Supplementary material for: Adverse events associated with the delivery of telerehabilitation: A scoping review protocol
Source: PLoS One. 2024 Feb 21;19(2):e0297908. doi: 10.1371/journal.pone.0297908 (PMC10880959; doi:10.1371/journal.pone.0297908)
Supplement: S2 Appendix — (DOCX) [file pone.0297908.s002.docx]

**S2 Appendix:**

**MEDLINE(R) ALL 1946 to June 22, 2023** **Search Strategy**

| **#** | **Searches** | **Results** |
| --- | --- | --- |
| 1 | Telemedicine/ | 37260 |
| 2 | Videoconferencing/ | 2320 |
| 3 | Remote Consultation/ | 5725 |
| 4 | or/1-3 | 42884 |
| 5 | exp Rehabilitation/ | 352248 |
| 6 | 4 and 5 | 3150 |
| 7 | rehab*.tw,kf. | 221905 |
| 8 | 4 and 7 | 1207 |
| 9 | rh.fs. | 208266 |
| 10 | 4 and 9 | 952 |
| 11 | (remote* or tele* or virtual* or "vr" or videoconferenc* or "video conferenc*").tw,kf. | 469375 |
| 12 | (5 or 7) and 11 | 18196 |
| 13 | or/6,8,10,12 | 20021 |
| 14 | Telerehabilitation/ | 981 |
| 15 | telerehab*.tw,kf. | 1934 |
| 16 | "e-rehab*".tw,kf. | 20 |
| 17 | or/13-16 | 20426 |
| 18 | Safety/ | 41951 |
| 19 | Patient Safety/ | 25400 |
| 20 | (safe or safety or safeties or safely or unsafe).tw,kf. | 1102853 |
| 21 | Patient Harm/ | 219 |
| 22 | (harm or harmed or harmful or harming or harms).tw,kf. | 165679 |
| 23 | (risk or risks).tw,kf. | 2880460 |
| 24 | Medical Errors/ | 17727 |
| 25 | (adverse* adj5 (effect* or event* or incident or incidents or outcome*)).tw,kf. | 553468 |
| 26 | (critical adj5 (event* or incident or incidents or outcome*)).tw,kf. | 22448 |
| 27 | (negativ* adj5 (effect* or event* or impact* or incident or incidents or mistake or mistakes or outcome*)).tw,kf. | 210509 |
| 28 | (therap* adj5 (accident* or error or errors or excessive or incident or incidents or mistake or mistakes or unnecessary or wrong)).tw,kf. | 7307 |
| 29 | (outcome* adj5 harm*).tw,kf. | 2906 |
| 30 | complication*.tw,kf. | 1187914 |
| 31 | ae.fs. | 1989850 |
| 32 | or/18-31 | 6197051 |
| 33 | 17 and 32 | 5212 |
| 34 | limit 33 to english language | 5033 |
| 35 | limit 34 to yr="2013 -Current" | 3857 |
