# Supplementary material for: Adverse events associated with the delivery of telerehabilitation: A scoping review protocol
Source: PLoS One. 2024 Feb 21;19(2):e0297908. doi: 10.1371/journal.pone.0297908 (PMC10880959; doi:10.1371/journal.pone.0297908)
Supplement: S3 Appendix — (DOCX) [file pone.0297908.s003.docx]

**S3 Appendix:**

**Extraction Form**

| **ALL AUTHORS** |  |
| --- | --- |
| **YEAR OF PUBLICATION (and pre/post COVID, pre-COVID defined as before March 11, 2020 by WHO)** |  |
| **POPULATION AND SAMPLE SIZE**  - sex  - age group  - origin based on country (and also developed vs less developed)  - primary diagnosis (also classify according to musculoskeletal, neurologic, cardiac, pulmonary, cancer, geriatric, pediatric rehab, etc.)  - surgical vs non-surgical cases? |  |
| **BRIEF NAME**  Provide the name or a phrase that describes the intervention. |  |
| **WHY** Describe any rationale, theory, or goal of the elements essential to the intervention. |  |
| **WHAT** |  |
| Materials: Describe any physical or informational materials used in the intervention, including those provided to participants or used in intervention delivery or in training of intervention providers. Provide information on where the materials can be accessed (e.g. online appendix, URL). |  |
| Procedures: Describe each of the procedures, activities, and/or processes used in the intervention, including any enabling or support activities. Include any materials provided e.g. telerehabilitation kit or starter kit with exercise equipment, devices to monitor vital signs, guard belt etc. Describe the specific mode/s of telerehabilitation delivery (synchronous: videocall, phone call, instant messaging, web-based such as using either virtual reality or augmented reality; asynchronous: text/ audio/ video messaging, e-mails, on-demand resources; hybrid: combination of any synchronous and asynchronous methods), and outcome measures (social/psychological/physical adverse events, including severity if available) Specify types of exercises delivered through telerehab.  Identify if accompanied with a reliable caregiver or completed independently. |  |
| **WHO PROVIDED** For each category of intervention provider (e.g. psychologist, nursing assistant), describe their expertise, background and any specific training given. |  |
| **HOW** Describe the modes of delivery (e.g. face-to-face or by some other mechanism, such as internet or telephone) of the intervention and whether it was provided individually or in a group. |  |
| **WHERE** Describe the type(s) of location(s) where the intervention occurred, including any necessary infrastructure or relevant features. |  |
| **WHEN and HOW MUCH** Describe the number of times the intervention was delivered and over what period of time including the number of sessions, their schedule, and their duration, intensity or dose. |  |
| **TAILORING** If the intervention was planned to be personalized, titrated or adapted, then describe what, why, when, and how. |  |
| **MODIFICATIONS** If the intervention was modified during the course of the study, describe the changes (what, why, when, and how). |  |
| **HOW WELL** Planned: If intervention adherence or fidelity was assessed, describe how and by whom, and if any strategies were used to maintain or improve fidelity, describe them. Actual: If intervention adherence or fidelity was assessed, describe the extent to which the intervention was delivered as planned. |  |
| **OUTCOME MEASURE** Describe adverse events, and categorize social/psychological/physical, including severity if available. |  |
